# Whole-Genome Sequencing of Leishmania infantum chagasi Isolates from Honduras and Brazil

**DOI:** 10.1128/MRA.00471-21

**Published:** 2021-12-02

**Authors:** Fernando Tobias Silveira, Edivaldo Costa Sousa Junior, Rodrigo Vellasco Silvestre, André Guilherme Costa-Martins, Kenny da Costa Pinheiro, Wilfredo Sosa Ochoa, Thiago Vasconcelos dos Santos, Patrícia Karla Ramos, Samir Casseb, Sandro Patroca da Silva, Concepción Zúniga Valeriano, Luciana Vieira Lima, Marliane Batista Campos, Vania Lucia da Matta, Claudia Maria Gomes, Gabriela Venicia Flores, Carmen Maria Pacheco, Carlos Eduardo Corbett, Helder Nakaya, Márcia Dalastra Laurenti

**Affiliations:** a Parasitology Department, Evandro Chagas Institute, Surveillance Secretary of Health, Ministry of Health, Ananindeua, Pará, Brazil; b Tropical Medicine Nucleus, Federal University of Pará, Belém, Pará, Brazil; c Virology Department, Evandro Chagas Institute, Surveillance Secretary of Health, Ministry of Health, Ananindeua, Pará, Brazil; d Computational System Biology Laboratory, São Paulo University, São Paulo, São Paulo, Brazil; e Microbiology School, National Autonomous University of Honduras, Tegucigalpa, Honduras; f Health Surveillance Department, University School Hospital, National Autonomous University of Honduras, Tegucigalpa, Honduras; g Arbovirology Department, Evandro Chagas Institute, Surveillance Secretary of Health, Ministry of Health, Ananindeua, Pará, Brazil; h Laboratory of Pathology of Infectious Diseases, Medical School, São Paulo University, São Paulo, São Paulo, Brazil; University of California, Riverside

## Abstract

This work reports on the whole-genome sequencing of Leishmania infantum chagasi from Honduras (Central America) and Brazil (South America).

## ANNOUNCEMENT

Until the end of the past century, Leishmania chagasi was regarded as the causal agent of American visceral leishmaniasis (1) but, after recent genomic evidence showing that L. chagasi and Leishmania infantum are similar, L. chagasi has come to be considered synonymous with L. infantum (2–4). However, in light of evidence about the original, enzootic cycle of Leishmania infantum chagasi in the New World (5–7), we describe here the whole-genome sequence of L. infantum chagasi from Honduras (Central America) and compare it with the sequence of the same parasite from Brazil (South America) and that of L. infantum from Europe available in GenBank.

The two *Leishmania* species isolates used in this work were (i) L. infantum chagasi MHOM/HD/2017/M32502/Amapala District/Honduras, which was isolated from a human case of nonulcerated cutaneous leishmaniasis according to procedures approved by the Research Ethics Committee of the Medical School of São Paulo University (CAAE protocol number 64223917.1.0000.006) (8), and (ii) L. infantum chagasi MCER/BR/1981/M6445/Salvaterra/Pará State/Brazil, which was isolated from a crab-eating fox (Cerdocyon thous), the wild reservoir (6). Both L. infantum chagasi isolates were grown at 25°C for 7 days in Schneider medium supplemented with 10% fetal bovine serum, 10 μg/ml 1% l-glutamine, and 100 IU/mL ampicillin. A 3-mL aliquot was collected and used for DNA extraction with the ReliaPrep genomic DNA (gDNA) miniprep system (Promega, Madison, WI, USA).

The total DNA quality was assessed using a NanoDrop 2000 spectrophotometer (Thermo Fisher Scientific, Waltham, MA, USA). The genomic library was prepared using the Nextera XT DNA sample preparation kit (Illumina, USA). The quality of the library was verified using a Bioanalyzer 2010 system (Agilent Technologies), and the library was sequenced on a HiSeq 2500 instrument (Illumina) with a 2 × 100-bp paired-end format sequencing kit v.4. The DNA concentration used for sequencing was 1 ng/μL in the Illumina HiSeq system. The reads generated were trimmed with Trimmomatic v.0.39 (9) and assembled using a *de novo* strategy with SPAdes v.3.12 (10). Genomes were manually curated, and the final genomes were compared with that of the L. infantum reference strain using Geneious v.8.1.9 (11). A molecular clock analysis was also performed to compare the origin and ancestry of these parasites, using BEAST v.1.10.4 with three independent runs and the strict Yule-coalescent model of epidemiological dispersion, with 100 million generations and with the DNA polymerase alpha subunit gene (a highly conserved genomic region related to the evolutionary process of *Leishmania* parasites). All tools were used with default parameters unless otherwise specified.

**FIG 1:**
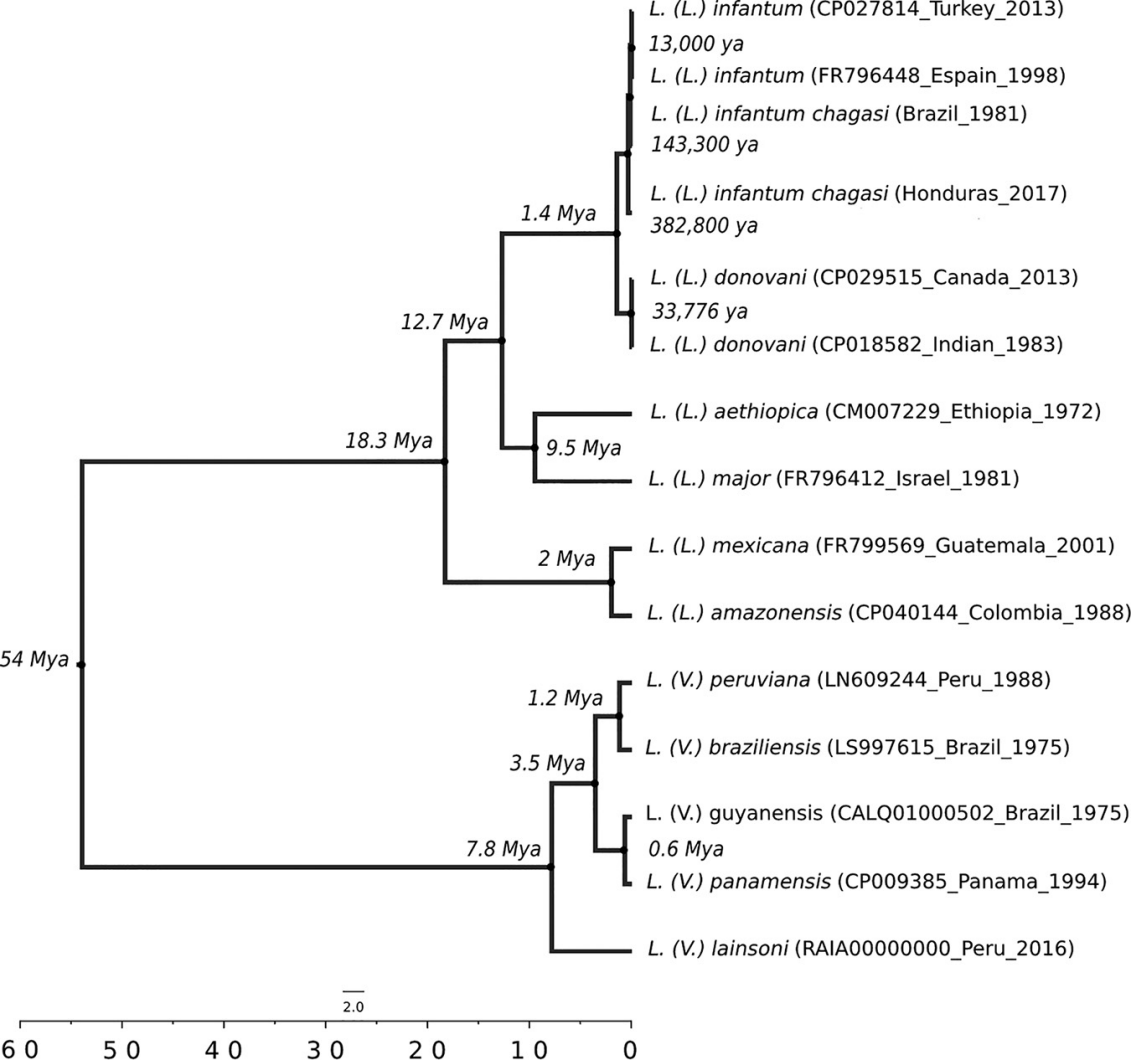
Bayesian divergence-time analysis under the relaxed molecular clock model for *Leishmania* species from the *Leishmania* and *Viannia* subgenera, using the concatenated data set for the DNA polymerase alpha subunit gene. The *x* axis shows absolute time in millions of years, and nodes are located at the mean divergence. The molecular clock analysis shows that New World Leishmania infantum chagasi isolates (Brazil_1981 [SRA accession number SRR8842312] and Honduras_2017 [SRA accession number SRR8608748]) experienced divergence ∼143,300 years ago (ya) and ∼382,800 years ago, respectively, and thus are more ancestral than L. infantum isolates from the Old World (Turkey_2013 and Espain_1998), with divergence ∼13,000 years ago. All nodes on the tree are supported with a posterior probability of 1.

The genomes of the L. infantum chagasi isolates from Honduras and Brazil showed ∼99.9% similarity to that of L. infantum when all genome chromosomes were compared (Table 1); however, the molecular clock comparisons revealed that L. infantum chagasi from Honduras proved to be considerably more ancestral (∼382,800 years ago) than L. infantum chagasi from Brazil (∼143,300 years ago) and L. infantum (∼13,000 years ago). In addition, it should also be emphasized that the DNA polymerase alpha subunit gene was able to reveal significant differences in ancestry between some *Leishmania* parasites belonging to the subgenera *Leishmania* and *Viannia* (Fig. 1).

**TABLE 1:** *De novo* genomic assembly of L. infantum chagasi isolates from Brazil and Honduras and their identity comparisons with L. infantum (from Europe)

Isolate	No. of contigs	Minimum size (bp)	Maximum size (bp)	*N*_50_ (bp)	Coverage (×)	GC content (%)	Identity (%)
MCER/BR/1981/M6445^*a*^	15,315	200	60,666	8,354	130	56.9	99.99
MHOM/HD/2017/M32502^*b*^	7,193	200	57,312	8,724	53.47	59.3	99.98

a L. infantum chagasi isolate from Brazil.

b L. infantum chagasi isolate from Honduras.

### Data availability.

The *de novo* whole-genome assemblies and raw data for Leishmania infantum chagasi from Brazil and Honduras have been deposited in the GenBank and SRA databases. For Leishmania infantum chagasi from Brazil, the GenBank accession number is JAGRQE000000000 and the SRA accession number is SRR8842312. For Leishmania infantum chagasi from Honduras, the GenBank accession number is JAGRQD000000000 and the SRA accession number is SRR8608748. Both are under BioProject number PRJNA722301.
